# A Dictionary Learning Method with Total Generalized Variation for MRI Reconstruction

**DOI:** 10.1155/2016/7512471

**Published:** 2016-03-15

**Authors:** Hongyang Lu, Jingbo Wei, Qiegen Liu, Yuhao Wang, Xiaohua Deng

**Affiliations:** Department of Electronic Information Engineering, Nanchang University, Nanchang 330031, China

## Abstract

Reconstructing images from their noisy and incomplete measurements is always a challenge especially for medical MR image with important details and features. This work proposes a novel dictionary learning model that integrates two sparse regularization methods: the total generalized variation (TGV) approach and adaptive dictionary learning (DL). In the proposed method, the TGV selectively regularizes different image regions at different levels to avoid oil painting artifacts largely. At the same time, the dictionary learning adaptively represents the image features sparsely and effectively recovers details of images. The proposed model is solved by variable splitting technique and the alternating direction method of multiplier. Extensive simulation experimental results demonstrate that the proposed method consistently recovers MR images efficiently and outperforms the current state-of-the-art approaches in terms of higher PSNR and lower HFEN values.

## 1. Introduction

Magnetic Resonance Imaging (MRI) plays an essential role in medical diagnostic tool, which provides clinicians with important anatomical information in the absence of ionizing radiation. Despite its superiority in obtaining high-resolution images and excellent depiction of soft tissues and acting as a noninvasive and nonionizing technique, the imaging speed in MR is limited by physical and physiological constraints. Its long scanning time leads to artifacts caused by the motion of patient's discomfort. Therefore, it is necessary to seek for a method to reduce the acquisition time. However, the reduction of the acquired data which compromises with its diagnostic value may result in degrading the image quality. Considering these reasons, finding an approach for accurate reconstruction from highly undersampled *k*-space data is of great necessity for both quick MR image acquisition and clinical diagnosis [1].

Generally, the existing regularization approaches fall into two categories: the predefined transform and the adaptively learned dictionary. The first category of predefined transform methods is usually related to total variation and wavelet transform. For example, Ma et al. employed the total variation (TV) penalty and the wavelet transform for MRI reconstruction [2]. As for the TV regularization that only considers the first-order derivatives, it is well known that it can preserve shape edges but often leads to stair casing artifacts and results in patchy, cartoon-like images which appear unnatural. In [3–6], the total generalized variation (TGV) which involves high-order derivatives was proposed. This regularization preserves the high-order smoothness better. Actually, TGV is equivalent to TV in terms of edge preservation and noise removal, which can also be true of imaging situations where the assumption of what TV is based on is not effective. It is more precise in describing intensity variation in smooth regions and thus reduces oil painting artifacts while still being able to preserve sharp edges like TV dose [7, 8]. Recently, Guo et al. proposed an outstanding method that combines shearlet transform and TGV (SHTGV) [9], which is able to recover both the texture and the smoothly varied intensities while the other methods such as shearlet and TGV only models either return a cartoon image or lose the textures. SHTGV is able to preserve edges and fine features and provide more “natural-looking” images. Although this work has improved the reconstruction result, it is still an analytically designed dictionary, which can be considered as only forcing the reconstructed image to be sparse with respect to spatial differences, as well as having intrinsic deficiency and lacking the adaptability to various images [10–12].

The second type of regularization method exploits the nonlocal similarity and sparse representation. Most of the existing DL models adopt a two-step iterative scheme, in which on the one hand the sparse representation approximations are found with the dictionary fixed and on the other hand the dictionary is subsequently optimized based on the current sparse representation coefficients [13–15]. Numerical experiments have indicated that these data-learning approaches obtained considerable improvements compared to previous predefined dictionaries-based methods [16, 17]. For instance, Ravishankar and Bresler assumed every image patch has sparse representation and proposed an outstanding two-step alternating method named dictionary learning-based MRI (DLMRI) reconstruction [18]. The first step is for adaptively learning dictionary; another step is for reconstructing image from highly undersampled *k*-space data. Nevertheless, most of existing methods fail to consider the representation of the sharp edge, which may lead to the loss of fine details. Motivated by this deficiency, we prefer to use total generalized variation to compensate the insufficiency of DL based methods [16–18].

In the proposed work, we exploit the strengths of both total generalized variation and patch-based adaptive dictionary for MR image reconstruction. This idea is motivated by the proceeding work about dictionary learning-based sparse representation and the second-order total generalized variation regularization. The proposed algorithm integrates the TGV regularizer and dictionary learning, which recovers both edges and details of images and selectively regularizes different image region at different levels. The solution of the proposed algorithm is derived by the variable splitting technique and the alternating direction method of multiplier (ADMM) [19, 20], alternatively calculating the dictionary and sparse coefficients of image patches and estimating the reconstructed image. The main contribution of this paper is the development of a more accurate and robust method. Firstly, the introduction of adaptively learned dictionary alleviates the artifacts caused by the piecewise smooth assumption and allows an image with complex structure to be recovered accurately. Secondly, the total generalized variation is equipped with options to accommodate the high degrees of smoothness that involved higher order derivatives and is more appropriate to represent the regularities of piecewise smooth images.

The remainder of this paper is organized as follows. We start with a brief review on the applications of dictionary learning and total generalized variation in Section 2. The proposed model integrating the dictionary learning and the TGV regularizer is presented and solved in detail in Section 3. Many numerical simulation results are illustrated in Section 4 to show the superiority of the proposed method, using a variety of sampling schemes and noise levels. Finally, conclusion is given in Section 5.

## 2. Background and Related Work

In this section, we review some classical models and algorithms for image reconstruction in the context of sparse representation. After the dictionary learning model and the total generalized variation algorithm were briefly reviewed, the proposed algorithm dictionary learning with total generalized variation (DLTGV) algorithm was derived in detail by incorporating the dictionary learning into the plain TGV algorithm. The following notation conventions are used throughout the paper. Let *u* ∈ *ℂ* ^*n*×*n*^ be the underlying image reconstructed, and let *b* ∈ *ℂ* ^*Q*^ represent the undersampled Fourier measurements. The partially sampled Fourier encoding matrix *K* ∈ *ℂ* ^*Q*×*n*^2^^ projects *u* to *b* domain such that *b* = *Ku* + *ξ*, with the *ξ* error. MRI reconstruction problem is formulated as the retrieval of the vector *u* based on the observation *b* and given the partially sampled Fourier encoding matrix *K*.

### 2.1. Dictionary Learning Recovery Model

Besides predefined sparsifying transform, sparse and redundant representations of image patches based on learned dictionaries have drawn considerable attention in recent years. Adaptive dictionary updating can represent image better than preconstructed dictionary. Owing to its adaptability to various image contents, dictionary learning possesses strong capability in preserving fine structures and details for image recovery problems. The patch-based sparsity can efficiently capture local image structures and can potentially alleviate aliasing artifacts. Sparse coding and simple dictionary updating steps make the algorithm converge in small iterations. The sparse model *J*(*u*) = (*λ*_0_/2)[‖*A*Γ − *Ru*‖_2_^2^ + *λ*_1_‖Γ‖_1_] is denoted as the regularization term for MRI reconstruction and solves the objective function as follows:

(1) min Ju=Ku−b≤ξ.

Consequently, the present method solves the objective function (1) by reformulating it as follows:

(2) u=Arg minD,Γ ∑β2Ku−b22+λ02AΓ−Ru22+λ1Γ1,

where *A* = [*a*_1_, *a*_2_,…, *a*_*J*_] ∈ *ℂ* ^*M*×*J*^ and Γ = [*x*_1_, *x*_2_,…, *x*_*I*_] ∈ *ℂ* ^*J*×*I*^. *Ru* stands for the extracted patches. The MR image is reconstructed as a minimizer of a liner combination of two terms corresponding to the dictionary learning-based sparse representation and least square data fitting. The first term enforces data fidelity in *k*-space, while the second term enforces sparsity of image with respect to an adaptive dictionary. The parameter *λ*_0_ balances the sparse level of the image patches and the approximation error in the updating dictionary. The parameter *λ*_1_ balances the weight of coefficient. For many natural or medical images, the value of *λ*_1_ can be determined empirically with robust performance in our work. *J* = *T* · *M*, *T* denotes the overcompleteness factor of the dictionary. The classical method to solve model (2) is DLMRI, through a two-step alternating manner. DLMRI model has performed superiorly compared to those using fixed basis. We exploit DL techniques to be more effective and efficient by adding high-order regularization of image, which will be presented in Section 3.

### 2.2. Total Generalized Variation

Image reconstruction using method of TGV achieves better results in many practical situations. The TGV of order *k* is defined as follows:

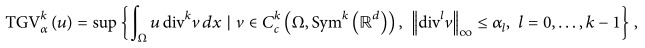
(3)where *C*_*c*_^*k*^(*Ω*, Sym^*k*^(*ℝ* ^*d*^)) is the space of compactly supported symmetric tensor field and Sym^*k*^(*ℝ* ^*d*^) is the space of symmetric tensor on *ℝ* ^*d*^. Choosing *k* = 1 and *α* = (1,1) yields the classical total variation. It constitutes a new image model which can be interpreted to incorporate smoothness from the first up to the *k*th derivative. Particularly, the second-order TGV can be written as

(4) TGVα2u=minp α1∇u−p1+α0ε−p1,

where directional derivatives ∇_1_ *u* and ∇_2_ *u* can be approximated by *D*_1_ *u* and *D*_2_ *u* and *D*_1_ and *D*_2_ are the circulate matrices corresponding to the forward finite difference operators with periodic boundary conditions along the *x*-axis and *y*-axis, respectively. Then ∇*u* is approximated by *Du* and $\overset{-}{\varepsilon }(p)$ is approximated by

(5) $$\begin{array}{c} \overset{-}{\varepsilon }\left(\;p\;\right)=\left[\begin{array}{cc} D_{1}p_{1} & \frac{1}{2}\left(D_{2}p_{1}+D_{1}p_{2}\right)\\ \frac{1}{2}\left(D_{2}p_{1}+D_{1}p_{2}\right) & D_{2}p_{2} \end{array}\right]. \end{array}$$

The proposed method derived another form of TGV_*α*_^2^ in terms of *l*_1_ minimization so that the model can be solved efficiently by ADMM. After discretization, (4) can be efficiently solved by ADMM. Image reconstruction with TGV regularization produces piecewise polynomial intensities. The convexity of TGV makes it computationally feasible. It refers to [3, 4] for further details and comparisons.

## 3. Proposed Algorithm DLTGV

In this work, we propose a new regularization scheme, combining adaptive dictionary learning with the regularization approach total generalized variation TGV_*α*_^2^ to reconstruct the target image with a lot of directional features and high-order smoothness. The dictionary learning is related to the image patch-based coefficient matrix and dictionary. The proposed method reconstructs the image simultaneously from highly undersampled *k*-space data and consists of a variable splitting solver alternating direction method of multiplier (ADMM). In the smooth regions of image *u*, the second derivative is locally small. Hence, using the generalized variation algorithm to regularize the nonconvex function will perform better, leading to a more faithful reconstruction of MR image. The proposed method recovers both edges and details of images and selectively regularizes different image region at different levels and thus largely avoids oil painting artifacts.

### 3.1. Proposed New Model

To reconstruct image *u* using the dictionary learning and total generalized variation regularization, we propose a new model to reconstruct the MRI images *u* as follows:

(6) minu β2Ku−b22+λ02AΓ−Ru22+λ1Γ1+TGVα2u,

where the parameter *β* > 0 is related to the noise level *ξ*. We utilize the second-order TGV_*α*_^2^ in our proposed method. With the new formulation of the discrete TGV_*α*_^2^ in (4), the proposed model (6) turns to be

(7) minu β2Ku−b22+λ02AΓ−Ru22+λ1Γ1+α1∇u−p1+α0ε−p1.

As in (5), the discrete version of (7) is

(8) minu β2Ku−b22+λ02AΓ−Ru22+λ1Γ1+α1Du−p1+α0ε−p1.

### 3.2. Algorithm to Solve Model (8)

As discussed in the previous section, dictionary updating and sparse coding to (2) are performed sequentially. In the following, we investigate that using TGV_*α*_^2^ as the regularization leads to an absence of the staircasing effect which is often observed in total variation regularization. To solve the proposed model, the first step of this alternating scheme is solved, image *u* is assumed fixed, and the dictionary and the sparse representations of the images are jointly updated. In the next step, the dictionary and sparse representation are fixed, and image *u* is updated through ADMM algorithm to satisfy data consistency.

The minimization equation (8) with respect to image *u* is derived as follows. Noting that there are two *l*_1_ terms in the reformulated model in (8) besides the second term, we apply ADMM to solve the optimization problem. We introduce auxiliary variable *y* and *z* for each *l*_1_ term:

(9) y=y1y2,z=z1z3z3z2.

So (8) is equivalent to

(10) minu,A,Γ β2Ku−b22+λ02AΓ−Ru22+λ1Γ1+α1y1+α0z1s.t y=Du−p, z=εp.

After applying the ADMM, we achieve the following algorithm:

(11) yn+1=arg miny y1+μ22y−Dun−pn−y~n22,zn+1=arg minz z1+μ32z−εpn−z~n22,An+1,Γn+1=arg minA,Γ AΓ−Run22+λ1Γ1,un+1,pn+1=arg minu,p λ02AΓ−Run22+β1μ22yn+1−Du−p−y~n22+β2μ32zn+1−εp−z~n22+β2Ku−b22,y~n+1=y~n+μDun+1−pn+1−yn+1,z~n+1=z~n+μεpn+1−zn+1.

Similar to the above section, we apply ADMM and decompose the optimization problem into five sets of subproblems as follows.

#### 3.2.1. Solve *y* ^*n*+1^ and *z* ^*n*+1^

The first two subproblems are similar and the solutions are given explicitly by shrinkage operation. The solution to the *y* subproblem is

(12) yn+1l=shrink2Dunl−pnl+y~nl,1μ2,l∈Ω,

where *y* ^*n*+1^(*l*) ∈ *ℝ* ^2^ represents the component of *y* ^*n*+1^ located at *l* ∈ *Ω*, and the isotropic shrinkage operator shrink_2_ is defined as

(13) $$\begin{array}{c} {\text{shrink}}_{2}\left(\;a,\mu \;\right)=\left\{\begin{array}{cc} 0, & a=0,\\ \left({\left\|a\right\|}_{2}-\mu \right)\frac{a}{{\left\|a\right\|}_{2}}, & a\neq 0. \end{array}\right. \end{array}$$

Likewise, we have the solution to the *z* problem as

(14) $$\begin{array}{c} z^{n+1}\left(\;l\;\right)={\text{shrink}}_{F}\left(\;\varepsilon \left(\;p^{n}\;\right)\left(\;l\;\right)+{\tilde{z}}^{n}\left(\;l\;\right),\frac{1}{\mu_{3}}\;\right),\;l\in \Omega , \end{array}$$

where *z* ^*n*+1^(*l*) ∈ *s* ^2×2^ is the component of *z* ^*n*+1^ corresponding to the pixel *l* ∈ *Ω* and

(15) $$\begin{array}{c} {\text{shrink}}_{F}\left(\;b,\mu \;\right)=\left\{\begin{array}{cc} 0, & b=0,\\ \left({\left\|b\right\|}_{F}-\mu \right)\frac{b}{{\left\|b\right\|}_{F}}, & b\neq 0. \end{array}\right. \end{array}$$

Note that 0 here is a 2 × 2 zero matrix and ‖·‖_*F*_ is the Frobenius norm of matrix.

#### 3.2.2. Update Dictionary and Coefficient

The minimization equation (8) with respect to dictionary and coefficient thus can be solved separately. Dictionary learning and coefficient updating step: in this step, the problem is solved with fixed image *u*, with the second term corresponding subproblem as follows:

(16) A,Γ=arg minA,Γ AΓ−Ru22s.t Γ1≤τ1.

The parameter *τ*_1_ in (16) is the required sparsity level. The strategy to solve (16) is to alternatively update dictionary *A* and sparsely represented coefficient Γ, the same as that used in K-SVD and DLMRI model. Specifically, in the sparse coding step, the solution of (16) is achieved by the orthogonal matching pursuit with respect to a fixed dictionary *A*. While at the dictionary updating step, the columns of the designed dictionary (represented by *a*_*k*_, 1 ≤ *k* ≤ *K*) are updated sequentially by using singular value decomposition (SVD) to minimize the approximation error. The K-SVD algorithm is used to learn the dictionary *A*. With the dictionary that is learnt, sparse coding is performed on the image to get the sparse represented coefficient Γ. Specifically, K-SVD is exploited to train the sparsifying dictionary for removing aliasing and noise, so that the target image *u* is reconstructed from learned dictionary and sparse representation.

#### 3.2.3. Solve *u* ^*n*+1^ and *p* ^*n*+1^

To solve the (*u*, *p*) subproblem, we obtain the second directional derivatives and the discretization with periodic boundary conditions, respectively, and then define the Lagrangian function. Taking the partial derivatives with respect to *u*, *p*_1_, *p*_2_, we get the normal equations as

(17) λ0AΓ−Ru+α1μ2∑j=12DjTDju−pj−yjn+1+y~jn+βK∗Ku−b=0,α1μ2p1−D1u+y1n+1−y~1n+α0μ3D1TD1p1−z1n+1+z~1n+12D2TD2p1+D1p2−2z3n+1+2z~3n=0,α1μ2p2−D2u+y2n+1−y~2n+α0μ3D2TD2p2−z2n+1+z~2n+12D1TD1p2+D2p1−2z3n+1+2z~3n=0.

Depending on the formulation of *K*, many methods can be used to solve (17) liner system. In this work, we illustrate the idea by means of solving the compressive sensing reconstruction problem. In this section, we fix attention on incomplete Fourier measurements as they have a wide range of applications in medical imaging and are very popular. We denote *K* = *F*_*p*_ = *PF*, where *P* is a selection matrix and *F* is a 2D matrix representing the 2D Fourier transform. The selection matrix *P* keeps the identity matrix if the data is sampled.

For incomplete Fourier transform, the subproblem equation (17) seems complicated. By the fact that it is easy to solve as the circulate matrix diagonalized by 2D Fourier transform *F*, next we demonstrate how to obtain the closed-form solution to (17). After grouping the like terms in (17), we get the following liner system:

(18) $$\begin{array}{c} \left[\begin{array}{ccc} d_{1} & d_{4}^{T} & d_{5}^{T}\\ d_{4} & d_{2} & d_{6}^{T}\\ d_{5} & d_{6} & d_{3} \end{array}\right]\left[\begin{array}{c} u\\ p_{1}\\ p_{2} \end{array}\right]=\left[\begin{array}{c} B_{1}\\ B_{2}\\ B_{3} \end{array}\right], \end{array}$$

where the block matrix is defined as

(19) d1=λ0I+α1∑j=12DjTDj+βK∗K,d2=α1μ2+α0μ3D1TD1+12D2TD2,d3=α1μ2+α0μ3D2TD2+12D1TD1,d4=−α1μD1,d5=−α1μD2,d6=12D1TD2,B1=λ0AΓ+α1μ2∑j=12DjTyjn+1−y~jn+βK∗b,B2=α1μ2y~1n−y1n+1+α0μ3D1Tz1n+1−z~1n+12D2T2z3n+1−2z~3n,B3=α1μ2y~2n−y2n+1+α0μ3D2Tz2n+1−z~2n+12D1T2z3n+1−2z~3n.

Next we multiply a preconditioner matrix from the left to linear system so that the coefficient matrix is block-wise diagonal:

(20) F000F000Fd1d4Td5Td4d2d6Td5d6d3F000F000F∗FuFp1Fp2=F000F000FB1B2B3.

The above operation can also be equivalently performed by multiplying each equation in (17) from the left with *F*. Denote

(21) d~1.⁡∗Fu+d~4T.⁡∗Fp1+d~5T.⁡∗Fp2=FB1,d~4.⁡∗Fu+d~2.⁡∗Fp1+d~6T.⁡∗Fp2=FB2,d~5.⁡∗Fu+d~6.⁡∗Fp1+d~3.⁡∗Fp2=FB3.

Similar to the scalar case, *Fu*, *Fp*_1_, and *Fp*_2_ can be obtained by applying the Cramer's rule. So *u*, *p*_1_, and *p*_2_ have the following closed forms:

(22) u=F∗FB1d~4Td~5TFB2d~2d~6TFB3d~6d~3∗./denom,p1=F∗d~1FB1d~5Td~4FB2d~6Td~5FB3d~3∗./denom,p2=F∗d~1d~4TFB1d~4d~2FB2d~5d~6FB3∗./denom,

where the division is component-wise and

(23) $$\begin{array}{c} \text{denom}={\left[\;\begin{array}{ccc} {\tilde{d}}_{1} & {\tilde{d}}_{4}^{T} & {\tilde{d}}_{5}^{T}\\ {\tilde{d}}_{4} & {\tilde{d}}_{2} & {\tilde{d}}_{6}^{T}\\ {\tilde{d}}_{5} & {\tilde{d}}_{6} & {\tilde{d}}_{3} \end{array}\;\right]}_{*}. \end{array}$$

Now, we summarize our proposed method for MRI reconstruction here, which we call dictionary learning with total generalized variation (DLTGV). The detailed description of the proposed method is listed in Algorithm 1. The proposed algorithm DLTGV alternatively updates image patch related coefficients, auxiliary variables, and the target solution *u*. The difference between the plain SHTGV and the DLTGV methods mainly lies on the difference of shearlet transform and dictionary learning. In DLTGV, adaptively learned dictionary alleviates the artifacts caused by the piecewise smooth assumption and allows an image with complex structure to be recovered accurately. The performance of DLTGV also depends on the selection of parameters, which will be explained in Section 4.

## 4. Experiments Results

In this section, the performance of proposed method was presented under a variety of sampling schemes and different undersampling factors. The sampling schemes used in our experiments include trajectory radial sampling, the 2D random sampling, and Cartesian sampling with random phase encoding (1D random). In the experiments, reconstruction results were obtained in simulated MRI data and complex-value data. The synthetic experiments used the images that are in vivo MR scans of size 512 × 512 (many of which are used in [18]). The complex-valued image [21, 22] in Figures 5 and 6 is of size 512 × 512 and those in Figures 7 and 8 are of size 256 × 256. According to many prior works on the CS data acquisition was simulated by subsampling the 2D discrete Fourier transform of the MR images (except the test with real acquired data).

In the experiments, our proposed method was compared with the leading DLMRI and SHTGV methods that have shown the substantial outperformance compared to other CS-MRI methods. The implementation coefficients of dictionary learning in DLMRI and our method DLTGV are the same, which is solved by K-SVD algorithm. The parameters of DLMRI and SHTGV methods were set to the default values. We introduced the peak signal-to-noise ratio (PSNR) and high-frequency error norm (HFEN) to quantify the quality of our reconstruction. All experiments were implemented in MATLAB 7.11 on a PC equipped with Intel core i7-3632QM and 4 GByte RAM.

### 4.1. Impact of Undersampling Schemes

**Figure 1:**
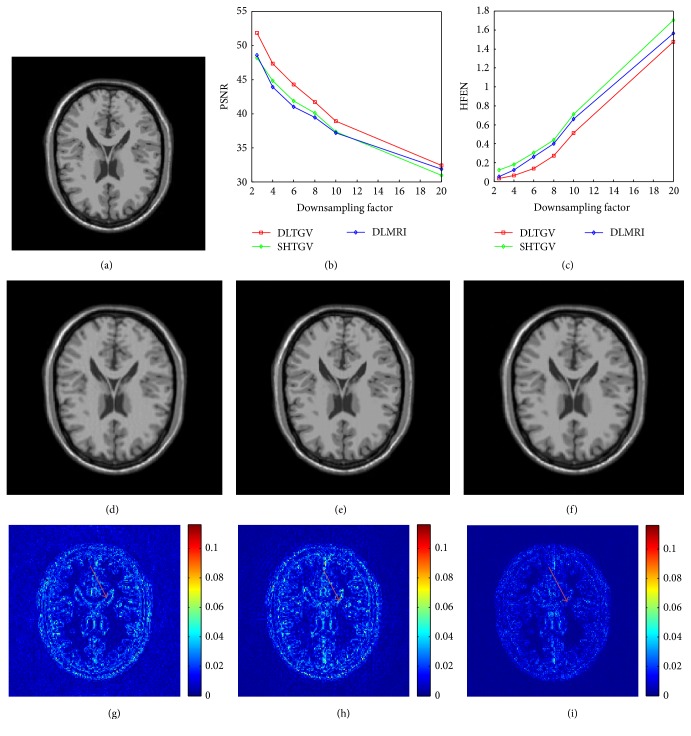
(a) The reference image. ((b), (c)) The PSNR and HFEN versus the downsampling factor. ((d), (e), (f)) The reconstruction results under pseudo radial sampling trajectory of DLMRI, SHTGV, and DLTGV. ((g), (h), (i)) The corresponding reconstruction error magnitudes of (d), (e), and (f).

In this subsection, we evaluated the performance of DLTGV under different undersampling ratio at pseudo radial sampling trajectory.  Figure 1 illustrates the reconstruction results with the pseudo radial sampled *k*-space at a range of undersampling factors with 2.5, 4, 6, 8, 10, and 20. The PSNR and HFEN values for DLMRI, SHTGV, and DLTGV at a variety of factors are presented in Figures 1(b) and 1(c). For the subjective comparison, the construction results and magnitude image of the reconstruction error produced by the three methods at 8-fold undersampling are presented in Figures 1(d), 1(e), and 1(f) and Figures 1(g), 1(h), and 1(i), respectively. As can be seen in Figure 1, the magnitude image of the reconstruction error for DLTGV shows less pixel errors and detail information than those of SHTGV (Figure 1(e)) and DLMRI (Figure 1(f), Table 1).

**Figure 2:**
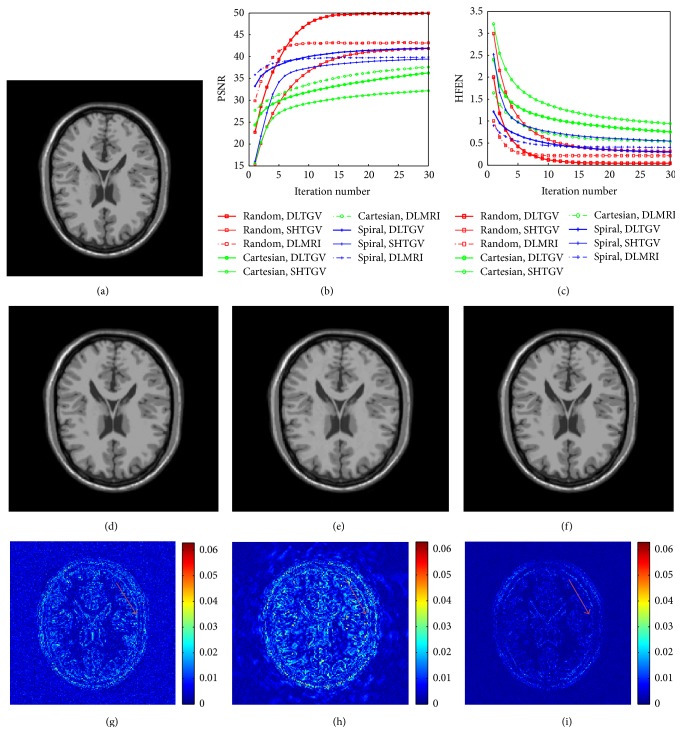
Reconstruction of an axial T2-weighed brain image at 7–11-fold undersampling. (a) The reference image. ((b), (c)) The PSNR and HFEN versus the number of iterations. ((d), (e), (f)) The reconstruction results under 2D random sampling by three methods DLMRI, SHTGV, and DLTGV. ((g), (h), (i)) The corresponding reconstruction error magnitudes of (d), (e), and (f).

The results with 7.11-fold undersampling of three different sampling schemes, including 2D random sampling, the sampling of central *k*-space phase encoding lines, and spiral sampling, are presented in Figure 2. The PSNR and HFEN curves are plotted in Figures 2(b) and 2(c) corresponding to DLMRI, SHTGV, and DLTGV. It can be seen that the more irrelevant the acquisition is, the better the reconstruction will be gained, and therefore the PSNRs obtained by 2D random sampling get more improvements than those of other sampling schemes. The results achieved by applying 2D random sampling are presented in Figures 2(d), 2(e), and 2(f). The magnitude error image for DLTGV shows that the reconstructed result using the proposed algorithm is more consistent than other methods. It can be seen that, under the same undersampling rate, the improvements gained by DLTGV outperform other methods at different trajectories.

### 4.2. Performance with Noise

**Figure 3:**
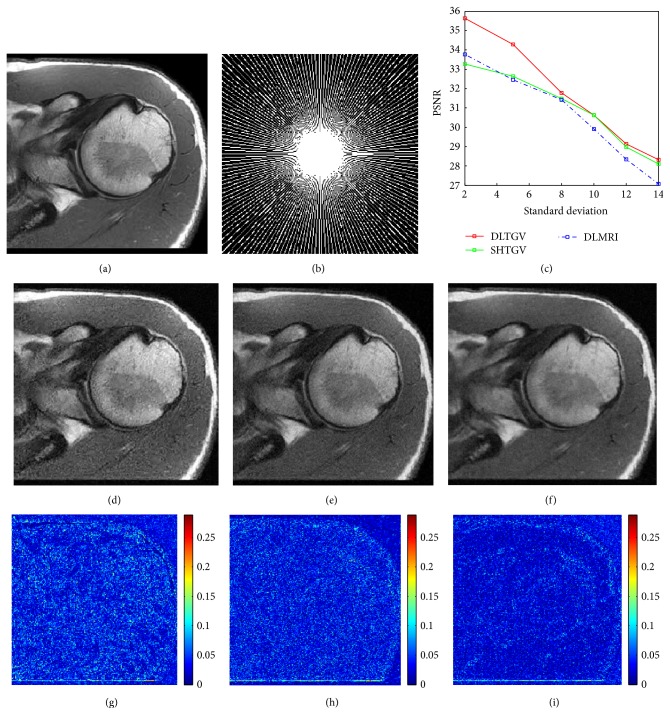
(a) Reference image. (b) Sampling mask in *k*-space with 6.09-fold undersampling. (c) PSNR versus noise level for DLMRI, SHTGV, and DLTGV. ((d), (e), (f)) Reconstructed images using DLMRI, SHTGV, and DLTGV. ((g), (h), (i)) Reconstruction error magnitudes for DLMRI, SHTGV, and DLTGV with noise *σ* = 14.

To investigate the sensitivity of DLTGV to different levels of complex white Gaussian noise, DLMRI, SHTGV, and DLTGV were applied to reconstruct image under pseudo radial sampling at 6.09-fold acceleration.  Figure 3 presents the reconstruction results of three methods at different levels of complex white Gaussian noise, which were added to the *k*-space samples. PSNRs of the recovered MR images by DLMRI (blue curves), SHTGV (green curves), and DLTGV (red curves) at a sequence of different standard deviations (*σ* = 2, 5, 8, 10, 12, 14) are shown in Figure 3(c). In the case of *σ* = 2, the PSNR of the image obtained by DLMRI is only 33.75 dB, SHTGV is 33.28 dB, and DLTGV reached 35.67 dB. Obviously, the difference gap between three methods is significant at low noise levels. The corresponding magnitudes of the reconstruction errors with *σ* = 14 are shown in Figures 3(d), 3(e), and 3(f). It can be observed that the DLTGV reconstruction appears less obscured than those in the DLMRI results. Meanwhile, the reconstruction by DLTGV is clearer than that by DLMRI and SHTGV and is relatively devoid of aliasing artifacts. It reveals that our method provides a more accurate reconstruction of image contrast and sharper anatomical depiction in noisy case.

### 4.3. Parameter Evaluation

**Figure 4:**
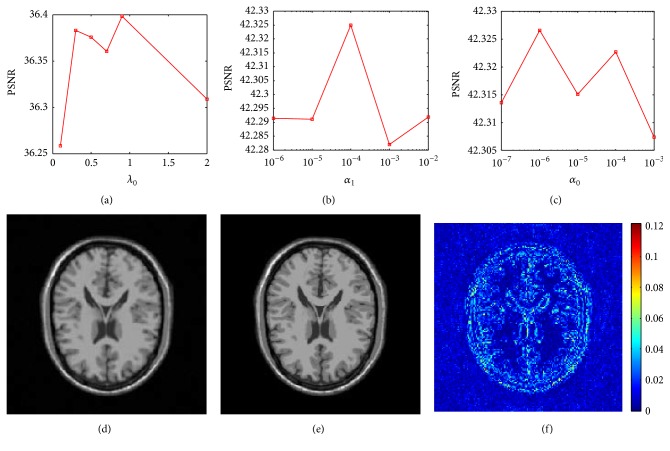
Parameter evaluation. (a) PSNR versus *λ*_0_. (b) PSNR versus *α*_1_. (c) PSNR versus *α*_0_. (d) The reference image. (e) Reconstructions with *λ*_0_ = 0.9, *α*_1_ = 10^−4^, and *α*_0_ = 10^−6^. (f) The reconstruction errors of (e).

Similar to the detail-preserving regularity scheme, this section evaluates the sensitivity of the proposed method to parameter settings by varying one parameter at a time while keeping the rest fixed at their nominal values. The parameter evaluation in Figure 4 was investigated in radial trajectory sampling with 8-fold undersampling. The parameters *β* and *λ*_1_ were observed to work well at their normal value and hence are not studied separately. The three parameters *λ*_0_, *α*_1_, and *α*_0_ are related to the noise level as well as the sparsity of underlying image of interest under dictionary leaning and TGV regularity. PSNRs values are plotted in Figure 4 over these parameters. It is obvious that more fine tuning of the parameters may lead to better results, but the results with the parameters setting are consistently promising. The plots of Figure 4 indicate that the “nominal” parameter values work reasonably well. The results demonstrate that the algorithm is not very sensitive to parameters and can be used without tuning.

### 4.4. Reconstruction of Complex-Valued Data

**Figure 5:**
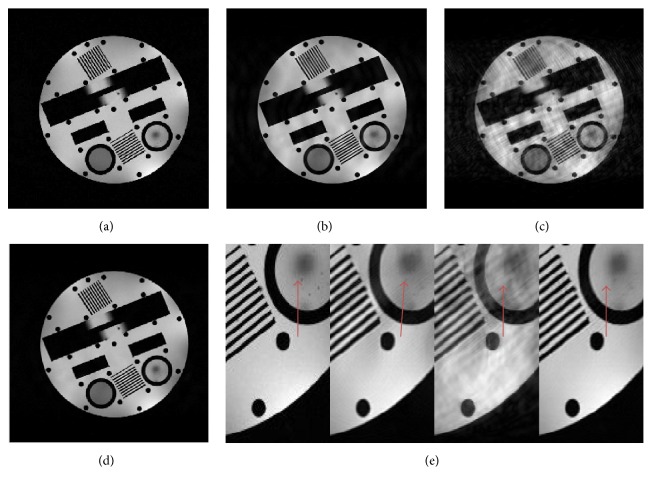
Reconstruction comparison of the physical phantom. (a) Fully sampled image. ((b), (c), (d)) Reconstruction results corresponding to DLMRI, SHTGV, and DLTGV at 8-fold undersampling. (e) The local area of enlargements of (a), (b), (c), and (d).

Figure 5 displayed the comparison results under Cartesian sampling on a physical phantom which is usually used to assess the resolution of MRI system. Figures 5(b), 5(c), and 5(d) showed the results of DLMRI, SHTGV, and DLTGV, at 8-fold undersampling. The PSNRs of DLMRI, SHTGV, and DLTGV were 29.02 dB, 22.78 dB, and 33.05 dB. Our result surpassed those of DLMRI and SHTGV, respectively, by 4.03 dB and 10.27 dB. The reconstruction with the three methods showed obvious differences in visual quality. While the DLMRI and SHTGV reconstructions displayed visible aliasing artifacts along the horizontal direction, the DLTGV reconstruction was more explicit and less artifacts at the same direction. The zoom-in map was presented in Figure 5(e).

**Figure 6:**
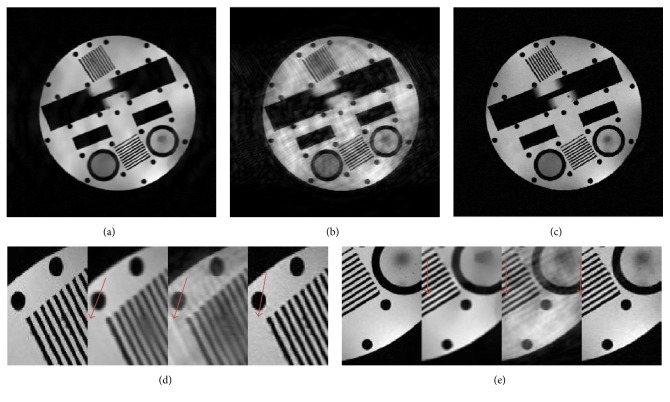
Comparison of reconstruction of a physical MR image with noise of *σ* = 30. ((a), (b), (c)) Reconstruction results of DLMRI, SHTGV, and DLTGV at 8-fold undersampling. ((d), (e)) The area of enlargements corresponding to (a), (b), and (c).

Furthermore, we added the noise *σ* = 30 to investigate the sensitivity of presented method on complex-valued data. The PSNR values of 26.33 dB, 22.00 dB, and 31.55 dB were obtained by DLMRI, SHTGV, and DLTGV, respectively. The reconstruction results of the three methods were shown in Figures 6(a), 6(b), and 6(c). The enlargements of two region-of-interests were presented in Figures 6(d) and 6(e). It indicates that the proposed method reflected the superior denoising ability compared to the other two methods. Moreover, the illustrated red arrow in Figures 6(d) and 6(e) showed that DLTGV exhibited less obscured phenomenon than that in the DLMRI and SHTGV results.

**Figure 7:**
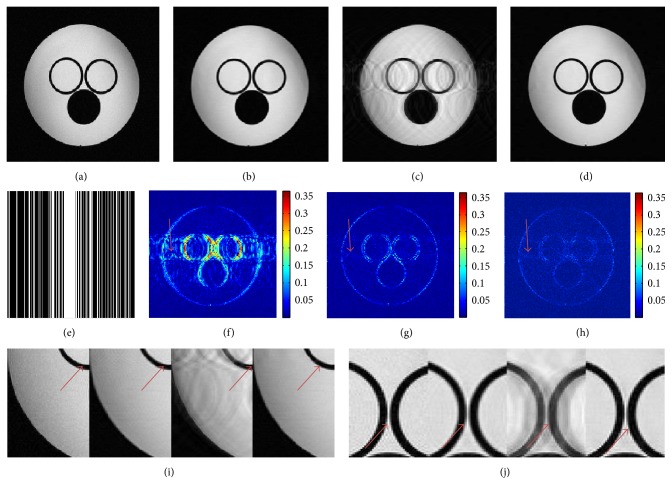
Reconstructed water phantom images at 35% undersampling. (a) The fully sampled image. ((b), (c), (d)) The reconstruction images corresponding to DLMRI, SHTGV, and DLTGV. (e) Mask data with 35% Cartesian sampling. ((f), (g), (h)) Reconstruction error magnitudes for DLMRI, SHTGV, and DLTGV. ((i), (j)) Enlargements of (a), (b), (c), and (d).

**Figure 8:**
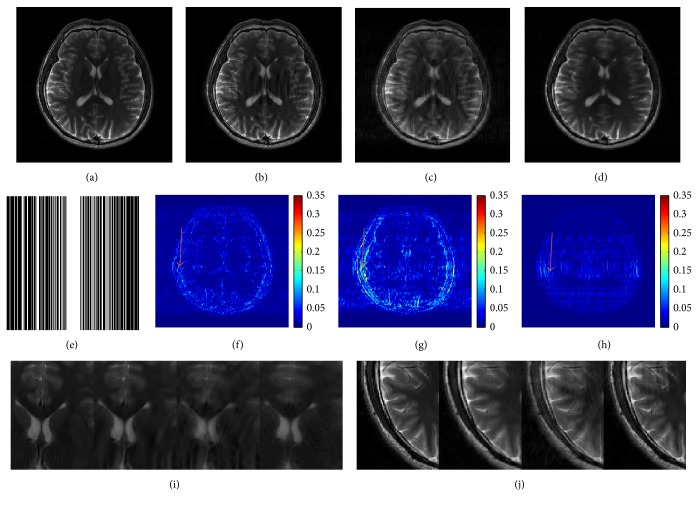
Reconstructed brain images at 40% undersampling. (a) The fully sampled image. ((b), (c), (d)) The reconstruction images using DLMRI, SHTGV, and DLTGV. (e) Mask data with 40% Cartesian sampling. ((f), (g), (h)) Reconstruction error magnitudes for DLMRI, SHTGV, and DLTGV. ((i), (j)) Enlargements of (a), (b), (c), and (d).

In order to further verify the performance of presented method DLTGV, we utilized the datasets [21, 22] which included complex-valued water phantom image in Figure 7(a) and T2-weighed brain image in Figure 8(a). For the water phantom image tested in Figure 7, Cartesian sampling trajectory with 35% undersampling was employed in this experiment. For the visual comparison, the proposed method produced better resolution and fewer artifacts than the other two methods. For the quantitative comparison, the PSNR values of DLMRI and SHTGV were 34.06 dB and 26.16 dB, and at the same time the PSNR value of DLTGV reached 36.56 dB. As can be observed in Figures 7(i) and 7(j), along the horizontal direction, the DLTGV reconstruction contained less aliasing artifacts than the other reconstructions.

The performance of using T2-weighed brain image was displayed in Figure 8. Cartesian sampling trajectory with 40% undersampling was employed. The PSNRs were 33.70 dB, 29.02 dB, and 35.16 dB obtained by DLMRI, SHTGV, and DLTGV, respectively. Figures 8(f) and 8(g) presented a microscopic comparison between the reference image and the results reconstructed by DLMRI, SHTGV, and DLTGV. It can be observed that the DLTGV has provided a better reconstruction of long object edge between tissues and suppressed aliasing artifacts. In general, the proposed method produced greater intensity fidelity to the image reconstructed from the full data.

## 5. Conclusion

In this paper, we proposed a novel algorithm based on adaptive dictionary learning and TGV regularization to reconstruct MR image simultaneously from highly undersampled *k*-space data. The TGV algorithm leads to better performance in the nonconvex function regularization and the dictionary learning is related to the image patch-based coefficient matrix and dictionary. To figure out the nondifferential terms in our model, we apply ADMM to solve the optimization problem. The whole algorithm converges in a small number of iterations by means of the accelerated sparse coding and simple dictionary updating. The proposed method recovers both edges and details of images and selectively regularizes different image region at different levels, thus largely avoiding oil painting artifacts. Numerical experiments show that the proposed method converges quickly and the performance is superior to other existing methods under a variety of sampling trajectories and *k*-space acceleration factors. Particularly, it achieves better reconstruction results than those by using SHTGV and DLMRI. It even provides highly accurate reconstructions for severely undersampled MR measurements.

## Figures and Tables

**Algorithm 1:**
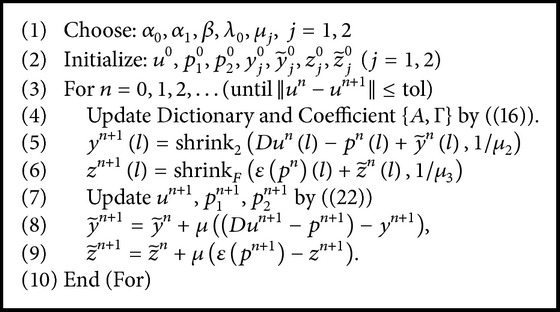
DLTGV.

**Table 1:** Reconstruction PSNR (dB) and HFEN values at different undersampling factors with the same pseudo radial sampling trajectories.

Downsampling factor	20-folder	10-folder	8-folder	6-folder	4-folder	2.5-folder
DLMRI	31.86 (1.56)	37.17 (0.66)	39.45 (0.40)	41.01 (0.26)	43.92 (0.12)	48.60 (0.05)
SHTGV	30.92 (1.70)	37.31 (0.71)	40.06 (0.44)	41.85 (0.31)	44.82 (0.18)	48.18 (0.12)
DLTGV	32.39 (1.48)	38.89 (0.51)	41.70 (0.27)	44.30 (0.14)	47.34 (0.06)	51.87 (0.03)
